# Prevalence of Carbapenem-Resistant Hypervirulent *Klebsiella pneumoniae* and Hypervirulent Carbapenem-Resistant *Klebsiella pneumoniae* in China Determined *via* Mouse Lethality Tests

**DOI:** 10.3389/fcimb.2022.882210

**Published:** 2022-06-01

**Authors:** Dakang Hu, Wenjie Chen, Qi Zhang, Meng Li, Zehua Yang, Yong Wang, Yunkun Huang, Gang Li, Dongxing Tian, Pan Fu, Weiwen Wang, Ping Ren, Qing Mu, Lianhua Yu, Xiaofei Jiang

**Affiliations:** ^1^ Department of Laboratory Medicine, Huashan Hospital, Fudan University, Shanghai, China; ^2^ Department of Infectious Diseases, Huashan Hospital, Fudan University, Shanghai, China; ^3^ Department of Laboratory Medicine, Henan Provincial People’s Hospital & the People’s Hospital of Zhengzhou University, Zhengzhou, China; ^4^ Department of Clinical Laboratory, The First Affiliated Hospital of Guangxi Medical University, Nanning, China; ^5^ Department of Laboratory Medicine, Sixth Hospital of Shanxi Medical University, Taiyuan, China; ^6^ Department of Clinical Laboratory, Shandong Provincial Hospital Affiliated to Shandong University, Jinan, China; ^7^ Department of Laboratory Medicine, Kunming Yan’an Hospital, Kunming, China; ^8^ Department of Laboratory Medicine, Jinshan Hospital of Fudan University, Shanghai, China; ^9^ Microbiology Department. Children’s Hospital of Fudan University, Shanghai, China; ^10^ Zhejiang Provincial Demonstration Centre of Laboratory Medicine Experimental Teaching, Wenzhou Medical University, Wenzhou, China; ^11^ School of Pharmacy, Fudan University, Shanghai, China; ^12^ Department of Laboratory Medicine, Taizhou Municipal Hospital, Taizhou, China

**Keywords:** carbapenem-resistant hypervirulent *Klebsiella pneumoniae*, hypervirulent carbapenem-resistant *Klebsiella pneumoniae*, epidemiology, mouse lethality test, hypervirulence, carbapenemase

## Abstract

**Objective:**

To investigate the epidemiology of carbapenem-resistant hypervirulent *Klebsiella pneumoniae* (CR-HvKP) and hypervirulent carbapenem-resistant *Klebsiella pneumoniae* (Hv-CRKP).

**Methods:**

Totally 436 *K. pneumoniae* strains were collected from 7 hospitals in mainland China between 2017.01 and 2018.02. Sequence types, serotypes, antimicrobial-resistance and virulence genes were analyzed. Additionally, string test, capsule stain, Periodic Acid Schiff stain, fitness analysis, quantitative real-time PCR and mouse lethality test were also performed. Molecular combinations were used to screen putative *bla*_KPC_(+)-HvKP and Hv-*bla*_KPC_(+)-KP, followed by the confirmation of mouse lethality test.

**Results:**

Diverse detection rates were found for the virulence genes, ranging from *c-rmpA* (0.0%) to *entB* (100.0%). According to the molecular criteria, 127, 186, 9 and 26 strains were putatively denoted as HvKP, *bla*_KPC_(+)-KP, *bla*_KPC_(+)-HvKP and Hv-*bla*_KPC_(+)-KP. Mouse lethality test confirmed 2 *bla*_KPC_(+)-HvKP strains (JS184 and TZ20) and no Hv-*bla*_KPC_(+)-KP. JS184 showed K2 serotype, thin capsule, positive exopolysaccharid and string test. TZ20 presented K20 serotype, thin capsule, negative exopolysaccharide and string test. Compared with the positive control NTUH-K2044, equal *galF* expression and growth curves were confirmed for JS184 and TZ20.

**Conclusions:**

Molecular determination of CR-HvKP and Hv-CRKP brings remarkable bias compared with mouse lethality test. The exact prevalence of CR-HvKP is less than 1.0%, which of Hv-CRKP is much lower.

## Introduction

*Klebsiella pneumoniae* is a gram-negative and rod-shaped bacterium that belongs to the *Enterobacteriaceae* family (Adeolu et al., 2016), and was first described by Carl Friedlander in 1882. *K. pneumoniae* is considered a prominent nosocomial pathogen worldwide, and is a member of the “ESKAPE” (*Enterococcus faecium*, *Staphylococcus aureus, K. pneumoniae*, *Acinetobacter baumannii*, *Pseudomonas aeruginosa*, and *Enterobacter* species) pathogens (Pendleton et al., 2013). General nosocomial infections caused by *K. pneumoniae* include pneumonia, bacteraemia, and urinary tract infections (UTIs) (Paczosa and Mecsas, 2016). The frequent use of antimicrobials has resulted in the development of carbapenem-resistant *K. pneumoniae* (CRKP) strains which first emerged in 1996 (Yigit et al., 2001). CRKP strains generally contain mobile genetic elements harbouring a variety of antimicrobial resistance genes, including beta-lactamase *K. pneumoniae* carbapenemase gene *(bla*_KPC_), New Delhi metallo-β-lactamase gene (*bla*_NDM_), and oxacillinase-48 gene (*bla*_OXA-48_) (Lee et al., 2016; Zhang et al., 2015), among which *bla*_KPC_ has been found to be shared in approximately 78.6% (44/56) (Lin et al., 2020) and 89.5% (34/38) of isolates in two studies (Lin et al., 2018). CRKP strains account for over 30.0% of *K. pneumoniae* strains and present great challenges in clinical practice (Effah et al., 2020). CRKP is associated with mortality rates of 34.7% (17/49) for pneumonia, 37.8% (34/90) for bacteraemia, and 7.4% (9/121) for UTI (Hauck et al., 2016). Furthermore, CRKP treatment is associated with a higher medical cost than that of carbapenem-susceptible *K. pneumoniae* (Huang et al., 2018). CRKP constitutes a major public health issue, especially in endemic countries (Karampatakis et al., 2016). Therefore, CRKP control is considered a priority by the World Health Organization (World Health Organization, 2017). CRKP is usually denoted as classical *K. pneumoniae* (cKP) regarding its virulence (Russo and Marr, 2019; Zhang et al., 2020).

Hypervirulence in *K. pneumoniae* represents another major concern. Hypervirulent *K. pneumoniae* (HvKP) was first reported to cause pyogenic liver abscess (PLA) and septic endophthalmitis in seven healthy individuals (Liu et al., 1986). HvKP, which has a considerably lower median lethal dose (LD_50_) than that of cKP in mouse model, generally produces various virulence factors such as hypercapsules, excessive siderophores, exopolysaccharides, and fimbriae (Paczosa and Mecsas, 2016; Russo and Marr, 2019). Apart from PLA, HvKP can also cause multiple invasive infectious diseases such as endogenous endophthalmitis, necrotising fasciitis, and meningitis, and the infection can undergo metastatic spread. PLA is endemic to East Asia and associated with a morbidity rate of 15.45 per 100,000 person-years in 2011 and a mortality rate of 8.2% (Chen et al., 2016; Siu et al., 2012). It has been estimated that 60% of endogenous endophthalmitis cases are associated with PLA caused by *K. pneumoniae* (Wong et al., 2000). Even with intravenous and intravitreal antimicrobial treatment, 89% of endophthalmitis cases show visual acuity of light perception or worse, and over 40% of affected eyes require evisceration or enucleation (Yang et al., 2007).

Recently, a combination of hypervirulence and extreme drug resistance has been reported in *K. pneumoniae*, thereby exacerbating the scarcity of effective treatments and resulting in high mortality (Zhang et al., 2015; Gu et al., 2018). The prevalence of infections caused by carbapenem-resistant hypervirulent *K. pneumoniae* (CR-HvKP) and hypervirulent carbapenem-resistant *K. pneumoniae* (Hv-CRKP) presents a global concern and a great challenge in clinical practice. However, the epidemiology of CR-HvKP and Hv-CRKP has not been extensively studied. The estimated prevalence of CR-HvKP/Hv-CRKP strains ranges among 5.0–15.0% among CRKP strains in mainland China, based on molecular determination or the *Galleria mellonella* (greater wax moth) lethality test (Zhang et al., 2020; Zhan et al., 2017). The gold standard method for evaluating the virulence of *K. pneumoniae* involves the use of mouse models, rather than the *G. mellonella* lethality test (Russo and MacDonald, 2020). Here, we analysed 436 clinical *K. pneumoniae* strains using molecular techniques and mouse lethality tests to elucidate the prevalence of CR-HvKP and Hv-CRKP strains.

## Materials and Methods

### *K. pneumoniae* Strains

In this study, 436 non-duplicate and consecutive *K. pneumoniae* isolates were collected from seven hospitals across six provinces in China (Huashan Hospital, 180 strains; Jinshan Hospital, 28 strains; Taizhou Municipal Hospital, 84 strains; The First Affiliated Hospital of Guangxi Medical University, 20 strains; Kunming Yan’an Hospital, 34 strains; Sixth Hospital of Shanxi Medical University, 60 strains; Shandong Provincial Hospital Affiliated to Shandong University, 30 strains) from January 2017 to February 2018. The 436 isolates were collected from diverse sources: 255 isolates (58.5%) were obtained from sputum samples, 98 isolates (22.5%) from urine samples, 29 isolates (6.7%) from blood samples, and 54 isolates (12.4%) were obtained from other sources. All the isolates were cultured on sheep blood agar plates and kept at -80°C prior to use. Identification of *K. pneumoniae* was performed using a matrix-assisted laser desorption/ionization time-of-flight mass spectrometry system (Bruker Daltonics Inc., Fremont, CA, USA) using the standard strains *P. aeruginosa* ATCC 27853, *K. pneumoniae* ATCC 700603, and *E. coli* ATCC 25922 as controls.

*K. pneumoniae* NTUH-K2044 (Accession number: **AP006725.1**) obtained from the Department of Internal Medicine, National Taiwan University Hospital, Taipei, Taiwan, is a typical hypervirulent *K. pneumoniae* serotype K1 strain (Fang et al., 2004). *K. pneumoniae* HS11286 (Accession number: **CP003200.1**) isolated from the Department of Laboratory Medicine, Huashan Hospital, Fudan University, Shanghai, China, is a *K. pneumoniae* serotype K47 strain containing *bla*_KPC_ and low virulence (Liu et al., 2012).

**Figure 1:**
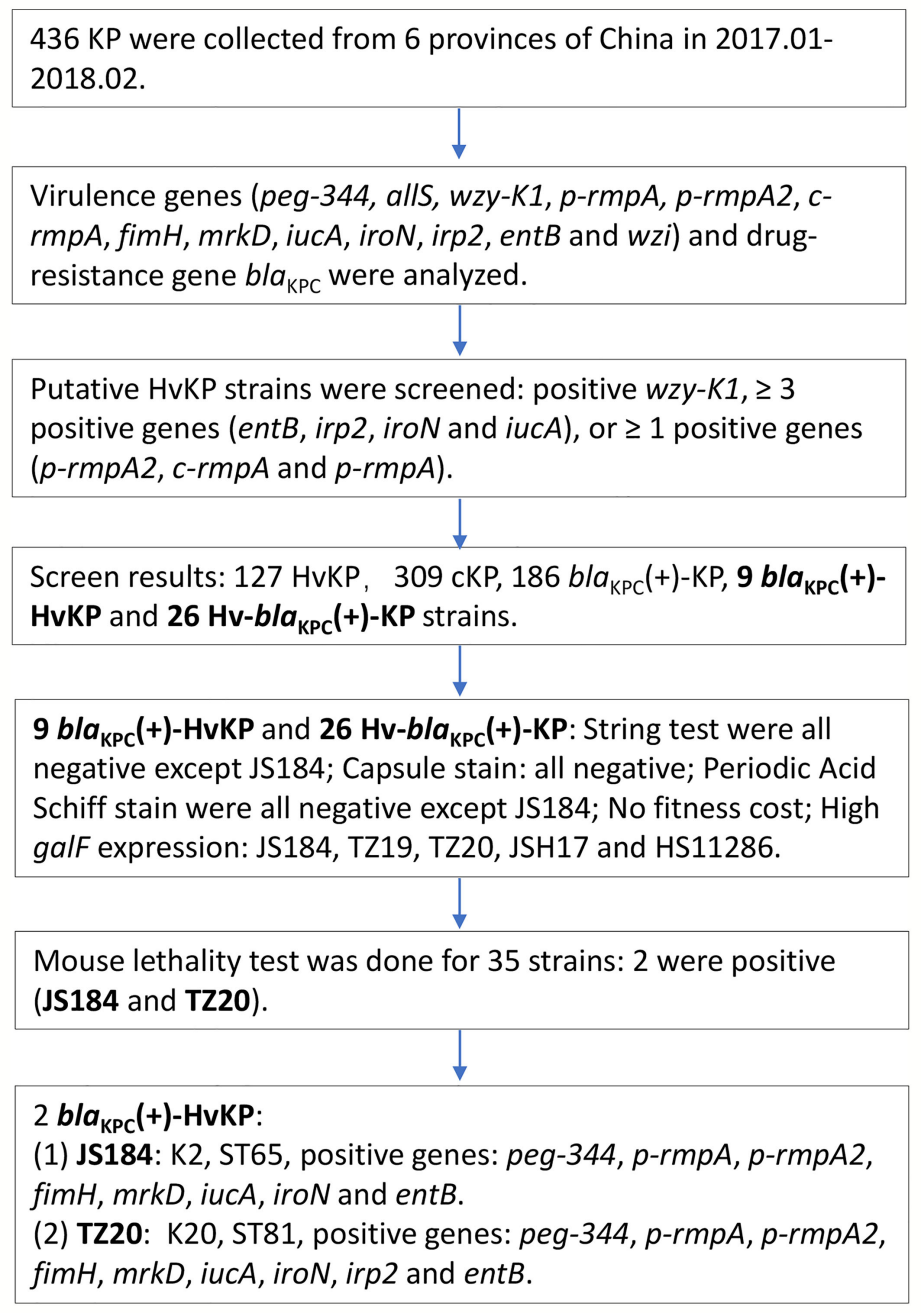
Experimental methodology of the study. KP, *Klebsiella pneumoniae*; HvKP, hypervirulent KP; *bla*_KPC_, beta-lactamase *K. pneumoniae* carbapenemase gene.

All the *K. pneumoniae* strains were investigated as the flow chart in **Figure 1**.

### Multilocus Sequence Typing

DNA was extracted from the 436 *K. pneumoniae* strains using the QIAamp DNA mini kit (QIAGEN, Düsseldorf, Germany) according to the manufacturer**’**s protocol. Seven housekeeping genes (*gapA*, *infB*, *mdh*, *pgi*, *phoE*, *rpoB*, and *tonB*) were amplified *via* polymerase chain reaction (PCR) (Diancourt et al., 2005) and then sequenced using an ABI 3730XL DNA Analyser (Applied Biosystems, San Ramon, CA, USA), and then compared with sequences available on the *K. pneumoniae* MLST database (http://www.pasteur.fr/recherche/genopole/PF8/mlst/Kpneumoniae.html). The primers used are shown in **Table S1**.

### Determination of Serotypes, Antimicrobial-Resistance, and Virulence Genes

The capsule type was determined *via* PCR amplification and sequencing of the *wzi* loci (Brisse et al., 2013), followed by comparison with sequences on the database of Institute Pasteur (https://bigsdb.pasteur.fr/klebsiella/klebsiella.html).

The antimicrobial resistance gene (*bla*_KPC_) and virulence genes (*wzy-K1*, *allS*, *entB*, *irp2*, *iroN*, *iucA*, *fimH*, *mrkD*, *p-rmpA2*, *c-rmpA*, *p-rmpA*, *peg-344*, and *wzi*) (Compain et al., 2014; Gu et al., 2018; Russo et al., 2018) were analysed *via* PCR amplification, using an Applied Biosystems Veriti PCR system (Applied Biosystems). The primers used are shown in **Table S1**.

### Determination of Putative HvKP, cKP, Hv-*bla*_KPC_(+)-KP, and *bla*_KPC_(+)-HvKP Strains

On the basis of molecular characteristics, HvKP and cKP were putatively defined as a reference (Hu et al., 2021) (**Figure 1**). Hv-*bla*_KPC_(+)-KP was defined as *bla*_KPC_-positive cKP which acquired key virulence genes that conferred hypervirulence. *bla*_KPC_(+)-HvKP (K1, K2, K5, K10, K20, K25, K27, and K57) (Chen et al., 2016; Siu et al., 2012) was defined as HvKP that acquired a *bla*_KPC_ gene.

### String Test

Overnight cultured *K. pneumoniae* colonies on sheep blood agar plates were stretched outward using an inoculation loop as described previously (Shon et al., 2013). The string test was considered positive when a viscous string produced was over 5 mm in length. Strain NTUH-K2044 was used as a positive control and HS11286 was used as a negative control.

### Capsule Staining

Capsule staining of *K. pneumoniae* strains was performed according to the manufacturer’s instructions (catalog number: BA-4039; BASO, Zhuhai, China). NTUH-K2044 was used as a positive control and HS11286 was used as a negative control.

### Periodic Acid-Schiff Staining

Periodic acid-Schiff staining was performed according to the manufacturer’s protocol (catalog number: BA4080A; BASO, Zhuhai, China). Strains NTUH-K2044 and HS11286 were used as positive and negative controls, respectively.

### Fitness Analysis

A growth curve was generated to evaluate the fitness of *K. pneumoniae* strains (Liu et al., 2016). These strains were cultured overnight in Luria-Bertani broth, diluted to an optical density at 600 nm (OD_600_) of 0.001, and cultured at 37°C under aerobic conditions (BioTek Synergy H1, Winooski, VT, USA). The OD_600_ values were measured every 30 min and plotted as a curve. Strains NTUH-K2044 and HS11286 were used as positive and negative controls, respectively.

### Quantitative PCR Analysis

Quantitative PCR analysis of *galF* mRNA together with 16S rRNA was performed using an Applied Biosystems 7500 system (Applied Biosystems, San Ramon, CA, USA). The primers used are shown in **Table S1**. Strains NTUH-K2044 and HS11286 were used as positive and negative controls, respectively. The analyses were performed according to the manufacturer’s protocol (catalog number: FS-Q1002; FOREVER STAR, Beijing, China).

### Mouse Lethality Test

Mouse experiments were approved by the Institutional Animal Care and Use Committee of the School of Pharmacy, Fudan University (Shanghai, China) (ethical approval document NO. 201603-TY-MQ-01). Pathogen-free female BALB/c mice (age, 6 weeks), four per group, were intraperitoneally inoculated with 100 μL of *K. pneumoniae* strains at the mid-logarithmic growth phase (Mizuta et al., 1983). Before inoculation, *K. pneumoniae* strains were washed twice with normal saline and centrifuged at 10,621 × *g* for 4 min. A 0.6 McFarland standard equivalent to 2.0 × 10^8^ colony forming units (CFU)/mL was prepared. The final inoculation was 10^2^–10^7^ CFU/mL. The mice were observed for 14 d after inoculation. LD_50_ was determined according to a previous study (Reed and Muench, 1938). Strains NTUH-K2044 and HS11286 were used as positive and negative controls. *K. pneumoniae* strains with LD_50_ ≤ 10 times of that of NTUH-K2044 were regarded as hypervirulent; those with LD_50_ > 10 times of that of NTUH-K2044 were denoted as hypovirulent.

### Statistical Analysis

GraphPad Prism 8 software (GraphPad Software Inc., Sand Diego, CA, USA) was used to perform Chi-square test, one-way ANOVA, and Kruskal-Wallis test between groups; *p* < 0.05 was considered significant.

## Results

### Distribution of Key Virulence Genes

**Figure 2:**
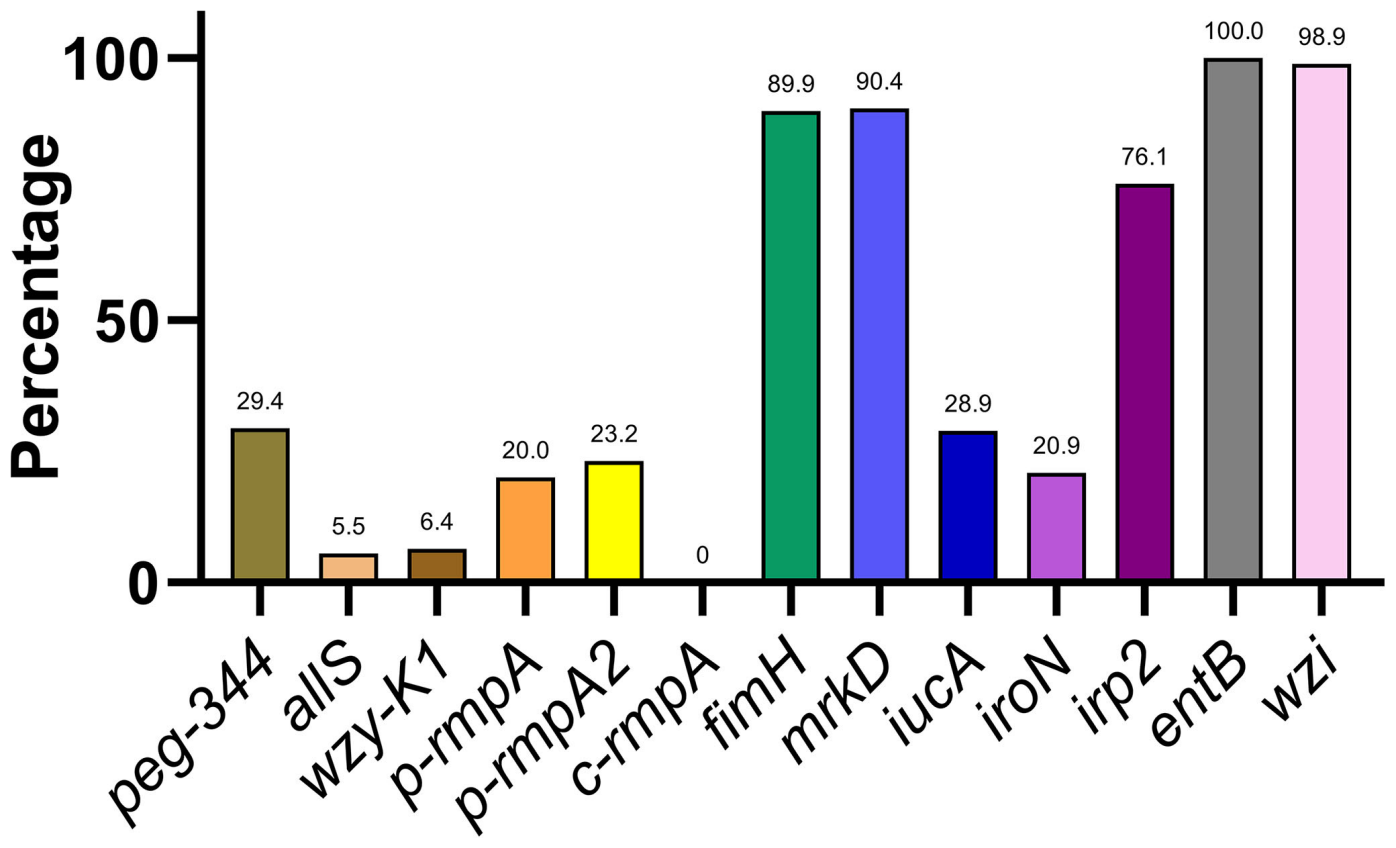
Distribution of 13 key virulence genes among the *Klebsiella pneumoniae* isolates.

A varying distribution of virulence genes was observed, ranging from 0.0% (*c-rmpA*) to 100.0% (*entB*) (**Figure 2**). The 13 virulence genes could be classified into 4 categories based on rates of distribution: ≤ 10.0% (*allS*, *wzy-K1*, and *c-rmpA*), approximately 11.0–30.0% (*peg-344*, *p-rmpA*, *p-rmpA2*, *iucA*, and *iroN*), approximately 50.0–80.0% (*irp2*), and approximately 81.0–100.0% (*fimH*, *mrkD*, *entB*, and *wzi*).

### Distribution of Predicted HvKP, *bla*_KPC_(+)-KP, *bla*_KPC_(+)-HvKP, and Hv-*bla*_KPC_(+)-KP

In total, 127 (29.1%), 186 (42.7%), 9 (2.1%), and 26 (6.0%) strains were putatively denoted as HvKP, *bla*_KPC_(+)-KP, *bla*_KPC_(+)-HvKP, and Hv-*bla*_KPC_(+)-KP strains, respectively.

### Distribution of Key Virulence Genes in Putative *bla*_KPC_(+)-HvKP and Hv-*bla*_KPC_(+)-KP

**Figure 3:**
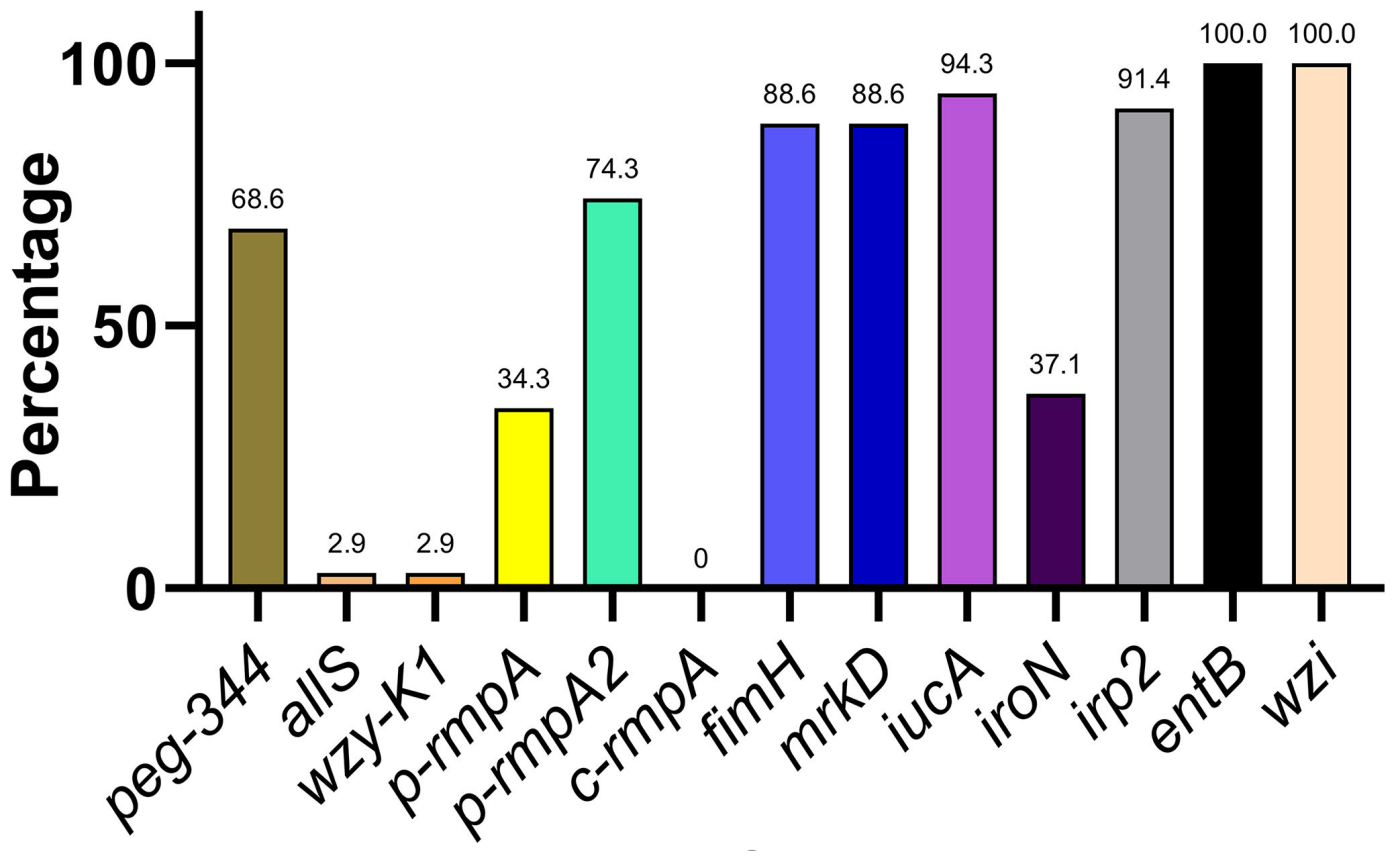
Distribution of key virulence genes among putative *bla*_KPC_(+)-HvKP and Hv-*bla*_KPC_(+)-KP strains. *bla*_KPC_, beta-lactamase *K. pneumoniae* carbapenemase gene; *bla*_KPC_(+)-HvKP, *bla*_KPC_(+) hypervirulent *K. pneumoniae*; Hv-*bla*_KPC_(+)-KP, hypervirulent *bla*_KPC_(+) *K. pneumoniae*.

The distribution of 13 key virulence genes among the putative *bla*_KPC_(+)-HvKP and Hv-*bla*_KPC_(+)-KP strains is shown in **Figure 3**. The 13 virulence genes were classified into four categories based on the rates of distribution: ≤ 10.0% (*allS*, *wzy-K1*, and *c-rmpA*), approximately 31.0–50.0% (*p-rmpA* and *iroN*), approximately 51.0–80.0% (*peg-344* and *p-rmpA2*), and approximately 81.0–100.0% (*fimH*, *mrkD*, *iucA*, *irp2*, *entB*, and *wzi*).

### Morphological Characteristics

**Figure 4:**
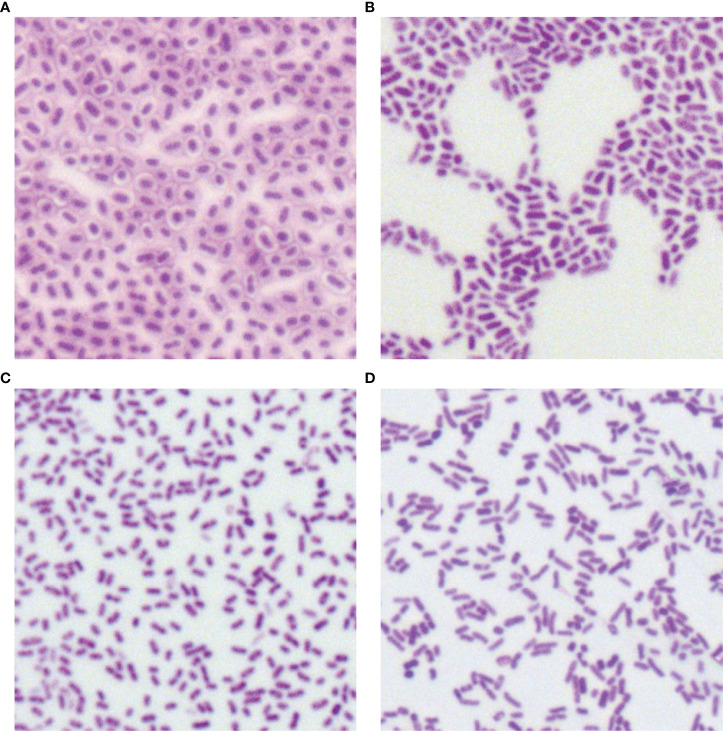
Capsule staining of 35 putative *bla*_KPC_(+)-HvKP and Hv-*bla*_KPC_(+)-KP strains. **(A)** *Klebsiella pneumoniae* NTUH-K2044, **(B)** *K. pneumoniae* HS11286, **(C)** JS184, and **(D)** TZ20. *K. pneumoniae* strains are purple and rod-shaped, and their transparent surroundings are hypercapsules (×1000). *bla*_KPC_, beta-lactamase *K. pneumoniae* carbapenemase gene; *bla*_KPC_(+)-HvKP, *bla*_KPC_(+) hypervirulent *K. pneumoniae*; Hv-*bla*_KPC_(+)-KP, hypervirulent *bla*_KPC_(+)* K. pneumoniae*.

**Figure 5:**
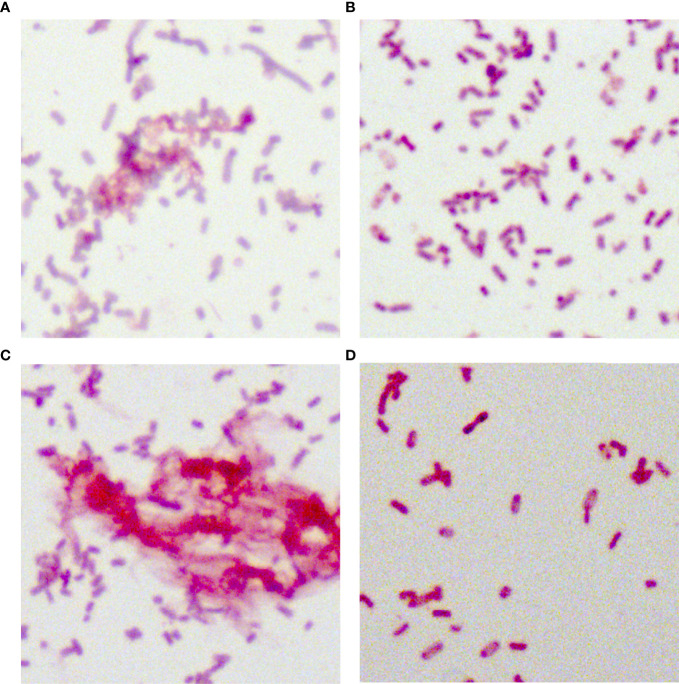
Periodic acid-Schiff staining of 35 putative *bla*_KPC_(+)-HvKP and Hv-*bla*_KPC_(+)-KP strains. **(A)** *Klebsiella pneumoniae* NTUH-K2044, **(B)** *K. pneumoniae* HS11286, **(C)** JS184, and **(D)** TZ20. *K. pneumoniae* strains were purple/red and rod-shaped; the red fluffy masses were exopolysaccharides. *bla*_KPC_, beta-lactamase *K. pneumoniae* carbapenemase gene; *bla*_KPC_(+)-HvKP, *bla*_KPC_(+) hypervirulent *K. pneumoniae*; Hv-*bla*_KPC_(+)-KP, hypervirulent *bla*_KPC_(+) *K. pneumoniae*.

In total, 34 putative *bla*_KPC_(+)-HvKP and Hv-*bla*_KPC_(+)-KP strains, except for *bla*_KPC_(+)-HvKP strain JS184, demonstrated negative string test results. No hypercapsule was found among the 35 putative *bla*_KPC_(+)-HvKP and Hv-*bla*_KPC_(+)-KP strains (**Figure 4**). No exopolysaccharides were found to be produced by the putative *bla*_KPC_(+)-HvKP and Hv-*bla*_KPC_(+)-KP strains, except for JS184 (**Figure 5**).

### Fitness Analysis

**Figure 6:**
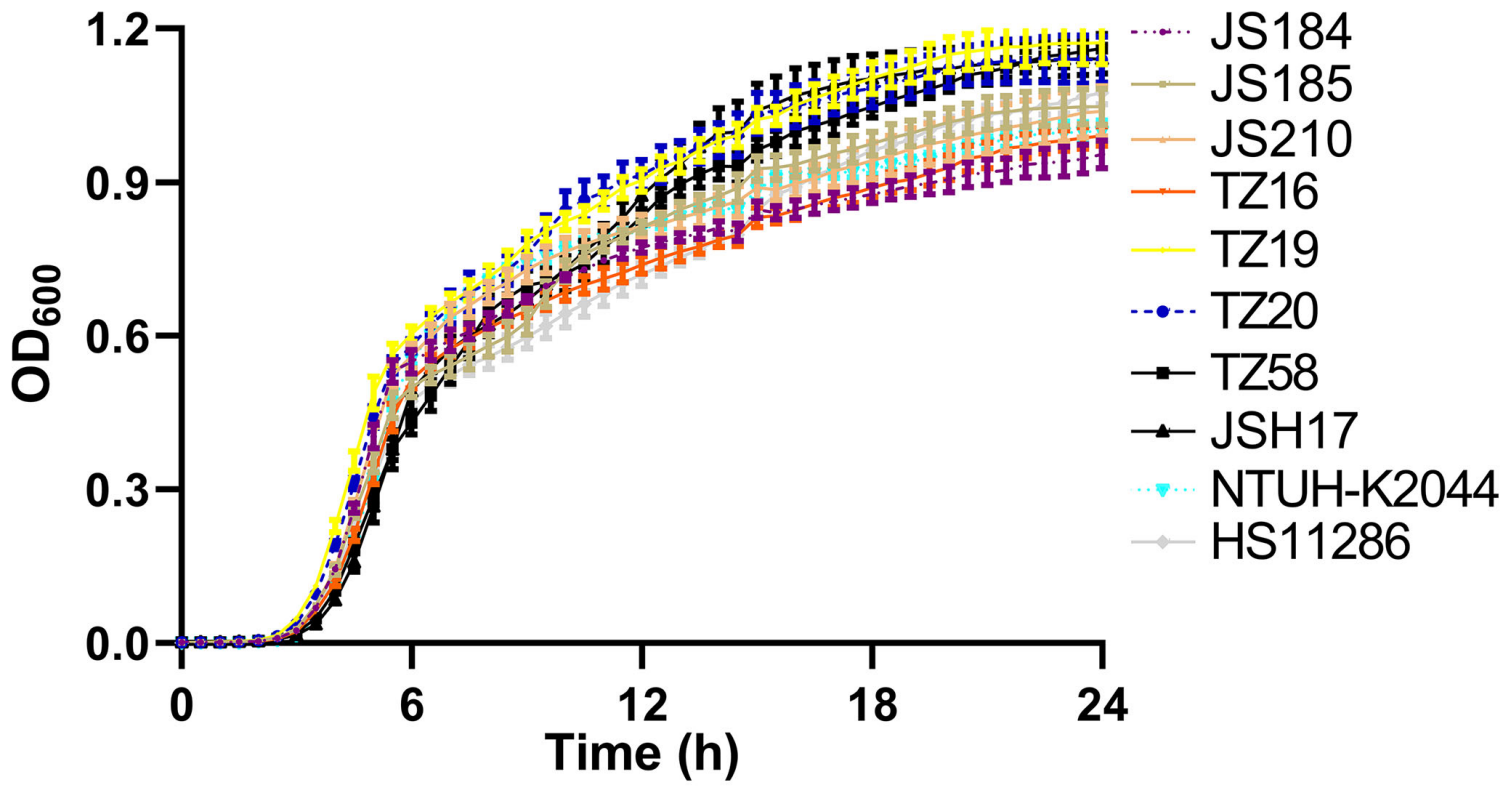
Growth curves of 10 putative *bla*_KPC_(+)-HvKP and Hv-*bla*_KPC_(+)-KP strains. *bla*_KPC_, beta-lactamase *K. pneumoniae* carbapenemase gene; *bla*_KPC_(+)-HvKP, *bla*_KPC_(+) hypervirulent *K. pneumoniae*; Hv-*bla*_KPC_(+)-KP, hypervirulent *bla*_KPC_(+) *K. pneumoniae*; OD_600_, optical density at 600 nm.

Among the 9 putative *bla*_KPC_(+)-HvKP and 26 Hv-*bla*_KPC_(+)-KP strains, 8 strains were chosen to represent each serotype: JS184 (K2, ST65), JS185 (K2, ST977), JS210 (K47, ST11), TZ16 (K64, ST11), TZ19 (K20, ST81), TZ20 (K20, ST81), TZ58 (K57, ST not defined), and JSH17 (K24, ST15). One-way ANOVA analysis indicated F = 0.9081 and *p* = 0.5178, which demonstrated similar growth and no fitness cost for the 8 strains (**Figure 6**).

### Expression of *galF*

**Figure 7:**
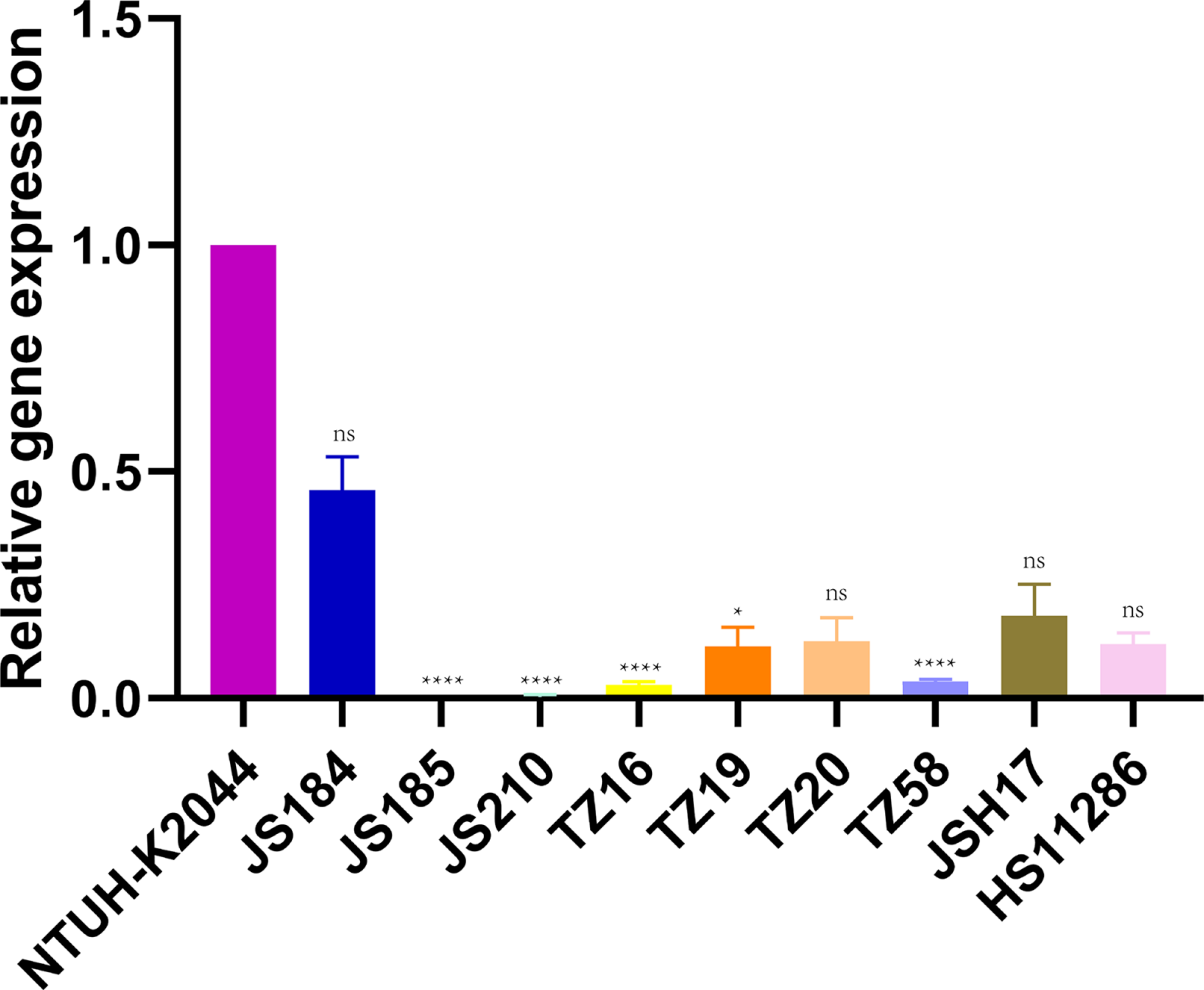
Expression of *galF* among 35 putative *bla*_KPC_(+)-HvKP and Hv-*bla*_KPC_(+)-KP strains. *Klebsiella pneumoniae* NTUH-K2044 was used as the standard to which others were compared. Kruskal-Wallis test was used for comparison. *bla*_KPC_, beta-lactamase *K. pneumoniae* carbapenemase gene; *bla*_KPC_(+)-HvKP, *bla*_KPC_(+) hypervirulent *K. pneumoniae*; Hv-*bla*_KPC_(+)-KP, hypervirulent *bla*_KPC_(+) *K. pneumoniae*; ns, not significant; ****, p < 0.0001; *, p < 0.05.

**Figure 7** shows the relative expression of *galF* in the putative strains compared to that in the control strains NTUH-K2044 and HS11286. JS184, TZ20, JSH17, and HS11286 showed high *galF* expression.

### Mouse Lethality Tests

**Figure 8:**
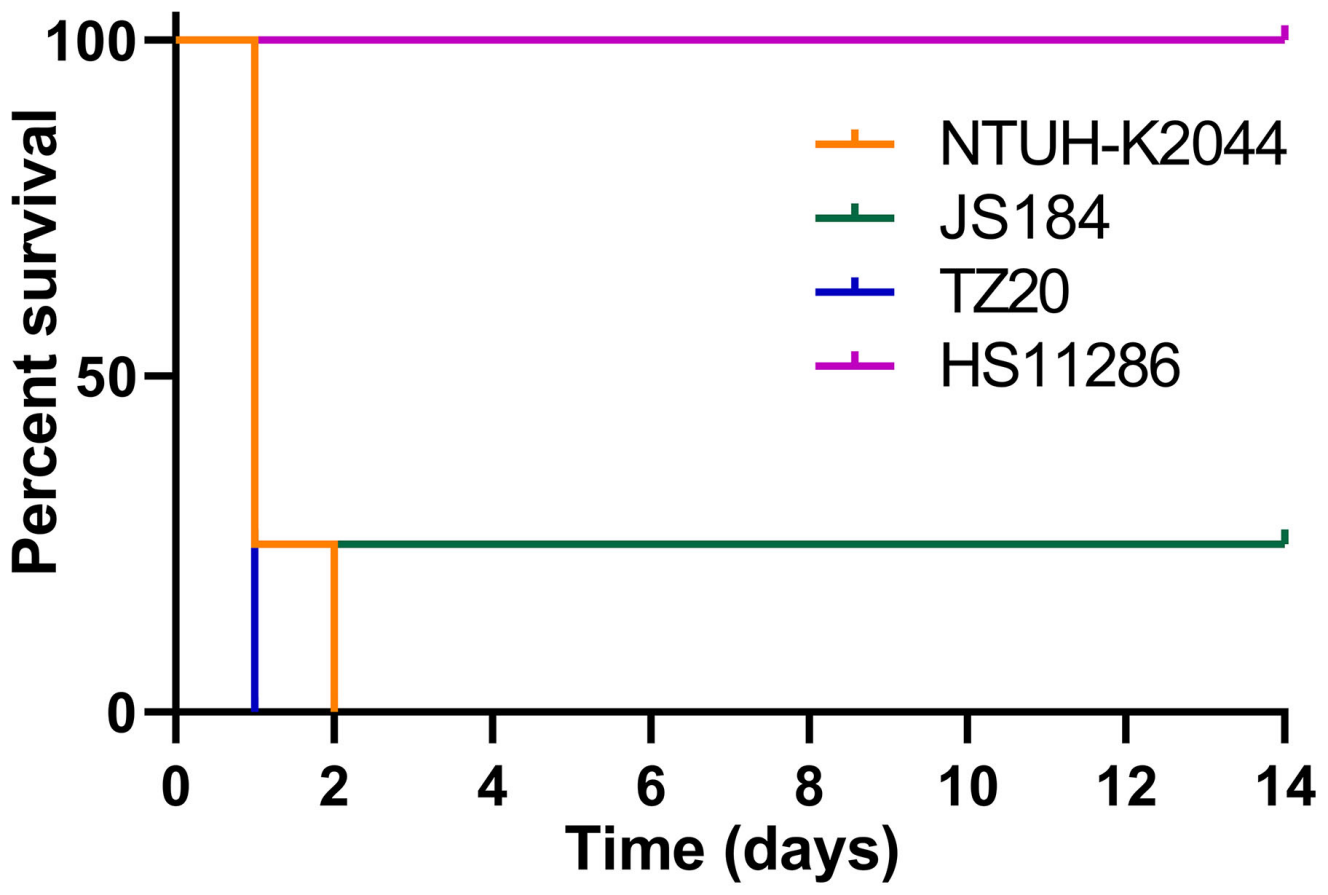
Survival curves of mice inoculated with two *bla*_KPC_(+)-HvKP strains. *bla*_KPC_(+)-HvKP, beta-lactamase *K. pneumoniae* carbapenemase gene-positive hypervirulent *Klebsiella pneumoniae*.

The survival curve for mice inoculated (10^6^ CFU) with the two *bla*_KPC_(+)-HvKP strains, JS184 and TZ20, is shown in **Figure 8**. Log-rank (Mantel-Cox) test yielded values of χ^2^ = 11.4286, *p* = 0.0096 for the four groups (JS184, TZ20, HS11286, and NTUH-K2044); χ^2^ = 1.5521, *p* = 0.4602 for three groups (JS184, TZ20, and NTUH-K2044). Therefore, the virulence of JS184 and TZ20 was similar to that of NTUH-K2044, and was higher than that of HS11286. The LD_50_ values were 10^6^ CFU for NTUH-K2044, 10^3^ CFU for JS184, < 10^6^ CFU for TZ20, > 10^7^ CFU for HS11286 and the other 33 putative *bla*_KPC_(+)-HvKP/Hv-*bla*_KPC_(+)-KP strains.

### Traits of Confirmed *bla*_KPC_(+)-HvKP Strains

The two confirmed *bla*_KPC_(+)-HvKP strains, JS184 and TZ20, showed differences in ST, *irp2* expression, and serotype (**Table 1**).

**Table 1:** Traits of confirmed *bla*_KPC_(+)-HvKP strains.

strain	ST	*peg-344*	*allS*	*wzy-K1*	*p-rmpA*	*p-rmpA2*	*c-rmpA*	*fimH*	*mrkD*	*iucA*	*iroN*	*irp2*	*entB*	serotype
JS184	65	+	–	–	+	+	–	+	+	+	+	–	+	K2
TZ20	81	+	–	–	+	+	–	+	+	+	+	+	+	K20

+, positive; -, negative; bla_KPC_(+)-HvKP, beta-lactamase Klebsiella pneumoniae carbapenemase gene-positive hypervirulent Klebsiella pneumoniae; ST, sequence type.

## Discussion

A combination of carbapenem resistance and hypervirulence in õõõõõ*K. pneumoniae* strains has been recently reported (Gu et al., 2018). However, the epidemiology of CR-HvKP and Hv-CRKP has not been extensively studied. To our knowledge, this is the first epidemiological surveillance study on *bla*_KPC_(+)-HvKP and Hv-*bla*_KPC_(+)-KP strains in China using a mouse lethality test to evaluate their prevalence.

In total, 13 key virulence genes in *K. pneumoniae* were investigated in this study; **Figure 2** shows a remarkable divergence in their distribution. The extremely high detection rates of *fimH*, *mrkD*, *entB*, and *wzi* indicate ubiquitous production of type 1 fimbriae, type 2 fimbriae, enterobactins, and capsules. Another siderophore gene *irp2* was present in a large proportion of the strains (76.1%), and is typically carried by ICE*Kp*1 in the chromosome; The remaining 2 siderophore genes *iucA* and *iroN* showed detection rates of approximately 20.0–30.0%, and are usually harboured by pK2044 and pLVPK-like virulence plasmids (Struve et al., 2015). The virulence genes *peg-344*, *p-rmpA*, and *p-rmpA2* are also present in pK2044- and pLVPK-like virulence plasmids (Russo and Marr, 2019), and therefore yielded similar detection rates to those of *iucA* and *iroN* (*p* > 0.05). The *c-rmpA* gene is present in the chromosome and often found in PLA specimens (Hsu et al., 2011). No such specimens were included in this study, which may explain the detection rate of 0.0%. The low detection rate of *wzy-K1* (6.4%) suggests the rarity of K1 *K. pneumoniae* in clinical practice.

Various molecular factors were evaluated to screen for CR-HvKP and Hv-CRKP. Zhang et al. previously evaluated *iucA*, *iroN*, *rmpA*, and *rmpA2* to check for the presence of virulence plasmids, and performed the *G. mellonella* lethality test to identify CR-HvKP, which yielded a rate of 5.2% (55/1052) for CR-HvKP (Zhang et al., 2020). We previously defined HvKP as: positive *wzy-K1*, ≥3 positive siderophore genes (*entB*, *irp2*, *iroN*, and *iucA*), or ≥1 positive capsule-regulating genes (*p-rmpA2*, *c-rmpA/A2*, and *p-rmpA*), and estimated a rate of 5.6% (29/521) for Hv-*bla*_KPC_(+)-KP (Shon et al., 2013), which was also applied in this study. Harada et al. defined HvKP as strains carrying virulence genes, *rmpA*, *rmpA2*, *iroBCDN*, *iucABCD*, or *iutA* (Harada et al., 2019). Russo et al. confirmed that the *G. mellonella* lethality experiment cannot accurately differentiate HvKP from cKP (Russo and MacDonald, 2020). Thus, the mouse lethality test may represent the only approach to determine the exact prevalence of CR-HvKP/Hv-CRKP. In this study, only two *bla*_KPC_(+)-HvKP strains, but no Hv-*bla*_KPC_(+)-KP strains, were eventually confirmed using a mouse lethality test, showing a rate of 0.5% (2/436) for CR-HvKP which was far lower than that reported in other studies (Zhang et al., 2020; Hu et al., 2021). Owing to the predominance of KPC-induced carbapenem resistance (Lin et al., 2018), the actual prevalence of CR-HvKP should be approximately 0.5–1.0% in mainland China in 2017. The considerable difference in prevalence rates determined between this study and other reports highlights the need to elucidate why such biomarkers are not reliable and the difference between mouse and *G. mellonella* lethality tests. Zhang et al., reported that only one *K. pneumoniae* strain has been confirmed as CR-HvKP among three strains that harbour *rmpA* based on a mouse lethality test (Zhang et al., 2015). The fact that *rmpA* genes are non-functional in cKP may be attributed to different genetic backgrounds, although *rmpA-*related genes, such as *kvrA*, *kvrB*, and *rcsB* (Palacios et al., 2018; Walker et al., 2019), were found to be widely distributed in both cKP and HvKP (data not shown).

Although Hv-CRKP and CR-HvKP strains are currently emerging worldwide (Gu et al., 2018; Karlsson et al., 2019), our study revealed that the emergence of CR-HvKP is a relatively greater concern owing to its prevalence. In this study, two confirmed *bla*_KPC_(+)-HvKP strains were found, including JS184 (K2) and TZ20 (K20), which showed no fitness cost, no hypercapsule production, and high expression of *galF* which is responsible for the synthesis of capsule precursor (Peng et al., 2018; Walker et al., 2019). However, JS184 showed a positive string test and exopolysaccharide production in contrast to TZ20. The reason for this is not known. In addition, JS184 and TZ20 also showed different ST and *irp2* expression.

This study had a few limitations. First, only typical siderophore genes were referred to, but not their expression. Second, capsule staining is not sufficient to differentiate capsules of various thicknesses, which may impact virulence.

Taken together, our findings indicate that CR-HvKP may emerge more often than Hv-CRKP; the former accounted for less than 1.0% of the strains evaluated *via* mouse lethality tests among clinical *K. pneumoniae* strains in mainland China in 2017.

## Data Availability Statement

The datasets presented in this study can be found in online repositories. The names of the repository/repositories and accession number(s) can be found in the article/**Supplementary Material**.

## Ethics Statement

The animal study was reviewed and approved by the Institutional Animal Care and Use Committee of the School of Pharmacy, Fudan University (Shanghai, China).

## Author Contributions

DH, WC, and QZ contributed to conception of the study. ML, LY, ZY, YW, YH, GL, and XJ collected and identified the strains. DH, WC, QZ, PF, DT, and WW performed PCR and MLST analyses, string tests, capsular staining, periodic acid-Schiff staining, and fitness tests. PR and QM performed mouse lethality tests. DH, WC, and QZ wrote the manuscript which was revised by XJ and LY. All authors read and approved the final manuscript.

## Funding

This study was supported by research grants from the National Natural Science Foundation of China (grants 81871692, 82172315, 82172315 and 81572031), Shanghai Municipal Key Clinical Specialty (Laboratory Medicine, No. shslczdzk03303), and the Shanghai Municipal Science and Technology Commission (grant number 19JC1413002).

## Conflict of Interest

The authors declare that the research was conducted in the absence of any commercial or financial relationships that could be construed as a potential conflict of interest.

## Publisher’s Note

All claims expressed in this article are solely those of the authors and do not necessarily represent those of their affiliated organizations, or those of the publisher, the editors and the reviewers. Any product that may be evaluated in this article, or claim that may be made by its manufacturer, is not guaranteed or endorsed by the publisher.
