# Supplementary material for: Prevalence of Carbapenem-Resistant Hypervirulent Klebsiella pneumoniae and Hypervirulent Carbapenem-Resistant Klebsiella pneumoniae in China Determined via Mouse Lethality Tests
Source: Front Cell Infect Microbiol. 2022 Jun 1;12:882210. doi: 10.3389/fcimb.2022.882210 (PMC9199425; doi:10.3389/fcimb.2022.882210)
Supplement: Supplementary file 1 [file Table_1.docx]

**Supplementary information**

Table S1 Primers used in this study

| Primer | Sequence (5′-3′) | Tm (℃) | Product (bp) |
| --- | --- | --- | --- |
| *rpoB* F | GGCGAAATGGCWGAGAACCA | 61 | 1,076 |
| *rpoB* R | GAGTCTTCGAAGTTGTAACC | 54 |  |
| *gapA* F | TGAAATATGACTCCACTCACGG | 58 | 663 |
| *gapA* R | CTTCAGAAGCGGCTTTGATGGCTT | 64 |  |
| *mdh* F | CCCAACTCGCTTCAGGTTCAG | 61 | 757 |
| *mdh* R | CCGTTTTTCCCCAGCAGCAG | 62 |  |
| *pgi* F | GAGAAAAACCTGCCTGTACTGCTGGC | 66 | 718 |
| *pgi* R | CGCGCCACGCTTTATAGCGGTTAAT | 66 |  |
| *phoE* F | ACCTACCGCAACACCGACTTCTTCGG | 69 | 603 |
| *phoE* R | TGATCAGAACTGGTAGGTGAT | 56 |  |
| *infB* F | CTCGCTGCTGGACTATATTCG | 58 | 463 |
| *infB* R | CGCTTTCAGCTCAAGAACTTC | 58 |  |
| *tonB* F | CTTTATACCTCGGTACATCAGGTT | 58 | 540 |
| *tonB* R | ATTCGCCGGCTGRGCRGAGAG | 65 |  |
| *wzy-K1* F | GGTGCTCTTTACATCATTGC | 55 | 1,283 |
| *wzy-K1* R | GCAATGGCCATTTGCGTTAG | 59 |  |
| *allS* F | CCGAAACATTACGCACCTTT | 57 | 508 |
| *allS* R | ATCACGAAGAGCCAGGTCAC | 60 |  |
| *entB* F | GTCAACTGGGCCTTTGAGCCGTC | 66 | 400 |
| *entB* R | TATGGGCGTAAACGCCGGTGAT | 65 |  |
| *irp2* F | GCTACAATGGGACAGCAACGAC | 62 | 230 |
| *irp2* R | GCAGAGCGATACGGAAAATGC | 60 |  |
| *iroN* F | GTCCGGCGGTAACTTCAGCC | 63 | 829 |
| *iroN* R | TCAGAATGAAACTACCGCCC | 58 |  |
| *iucA* F1 | AATCAATGGCTATTCCCGCTG | 59 | 239 |
| *iucA* R1 | CGCTTCACTTCTTTCACTGACAGG | 62 |  |
| *iucA* F2 | GCTTATTTCTCCCCAACCC | 56 | 583 |
| *iucA* R2 | TCAGCCCTTTAGCGACAAG | 57 |  |
| *fimH* F | TGCTGCTGGGCTGGTCGATG | 65 | 909 |
| *fimH* R | GGGAGGGTGACGGTGACATC | 62 |  |
| *mrkD* F | AAGCTATCGCTGTACTTCCGGCA | 64 | 340 |
| *mrkD* R | GGCGTTGGCGCTCAGATAGG | 64 |  |
| *p-rmpA2* F | GTGCAATAAGGATGTTACATTA | 52 | 230 |
| *p-rmpA2* R | GACTTATCATATTTAATGTT | 43 |  |
| *c-rmpA* F | GTAATAGAGATATAAATATCATATTGA | 49 | 589 |
| *c-rmpA* R | CATCTTTCATCAACCATTTC | 50 |  |
| *p-rmpA* F | GAGTAGTTAATAAATCAATAGCAAT | 52 | 332 |
| *p-rmpA* R | CAGTAGGCATTGCAGCA | 55 |  |
| *peg-344* F1 | CTTGAAACTATCCCTCCAGTC | 55 | 508 |
| *peg-344* R1 | CCAGCGAAAGAATAACCCC | 56 |  |
| *peg-344* F2 | AAAGGACAGAAAGCCAGTG | 55 | 411 |
| *peg-344* R2 | CAATGACGAGGGGGATAATC | 55 |  |
|  |  |  |  |
|  |  |  |  |
| *galF* F | GAAGAACGCGGTGGAAAACC | 60 | 131 |
| *galF* R | GCACGTTCATGATGGTCACG | 60 |  |
| *16S rRNA* F | TACCGCATAACGTCGCAAGA | 60 | 149 |
| *16S rRNA* R | TTCCAGTGTGGCTGGTCATC | 60 |  |
| *bla_KPC_* F | ATG TCA CTG TAT CGC CGTCT | 59 | 893 |
| *bla_KPC_* R | TTT TCA GAG CCT TAC TGCCC | 58 |  |

Tm, melting temperature.
